# The Impact of Bipolar Disorder on Couple Functioning: Implications for Care and Treatment. A Systematic Review

**DOI:** 10.3390/medicina57080771

**Published:** 2021-07-29

**Authors:** Jean-Michel Azorin, Antoine Lefrere, Raoul Belzeaux

**Affiliations:** Department of Psychiatry, Sainte Marguerite Hospital, 13009 Marseille, France; antoine.lefrere@ap-hm.fr (A.L.); raoul.belzeaux@ap-hm.fr (R.B.)

**Keywords:** bipolar disorder, marital relationship, couple relationship, couple functioning, partner, spouse, children, couple therapy

## Abstract

If there is an abundant literature on the impact of bipolar illness on the family and/or caregivers of patients, few studies have addressed its impact on marital relationship and couple functioning. Uncovering information relating specifically to this topic may be particularly relevant due to the unusually high divorce rate among individuals with bipolar disorder. We therefore conducted a systematic literature search to evaluate the existing data on bipolar disorder and marital issues, with a special focus on the help and support that can be provided by mental health professionals in this regard. We identified quantitative studies with pre-defined outcomes as well as qualitative investigations trying to understand the experiences of partners. A total of 27 articles were included in the review. The literature was found to capture the impact of bipolar disorder on partners as well as on the marital relationship itself or the children. Bipolar illness has a negative impact on the lives of partners including self-sacrifice, caregiver burden, emotional impact, and health problems. This negative impact can be aggravated by a lack of care and a lack of information from health personnel. The negative impact on the relationship includes volatility in the relationship, stigmatization, dissatisfaction with sexual life, and lower rates of childbearing. Negative impacts are likely to favor disease relapses for the patient. Children may also be negatively impacted. However, the illness may sometimes have positive impacts such as personal evolution, strengthening relationship, or new hope and perspectives. Based on these findings, the interventions of mental health professionals should be aimed at minimizing the negative impacts while favoring the positive ones.

## 1. Introduction and Methods

A couple relationship is defined as a relationship between partners characterized by substantial knowledge and understanding of one another, including physical intimacy and sexual involvement, in which partners are committed regardless of relationship status (cohabitation, civil partnership or marriage) [1].

Unlike cohabitation, civil partnership or marriage are based on legally recognized social contracts, which imply a permanence of the union and may give partners social and financial advantages. Union usually involves an alliance between individuals and/or families linked by a community of interests or feelings and can help for personal development and social career, by extending social network as well as providing affection and security to the partners. These unions also form the basis for procreation and building up of family [2].

Furthermore, in some countries like India, marriage is considered as a protective factor against mental illness and beliefs that marriage can cure mental illness are also quite prevalent [3]. In this regard, marital relationship could be viewed as a sort of ‘therapeutic alliance’. Bipolar disorder is a chronic mental disorder, which is one of the world’s 10 most disabling conditions [4]. Patients with bipolar disorder have a higher risk of committing suicide than patients with other psychiatric or medical disorders [5]. They also display an increased number of various psychiatric and somatic comorbidities [5].

The illness impacts many aspects of the patient’s life, such as employment, financial functioning, and social interactions [6].

In the United States, divorce and separation were also found to be two to three times more likely among bipolar patients than in the general population [7,8].

Many other studies [9,10,11] conducted in different countries support the findings of shorter duration of marriage among bipolar patients.

Although no ‘critical period’ was identified in the course of bipolar disorder that may impact more couple functioning, it was found that divorce was related to more hospitalizations, more severe episodes, and the presence of residual symptoms in between the episodes [2].

However, if there is an abundant literature on the impact of bipolar illness on the family and/or caregivers of patients, only a few studies have addressed its impact on marital relationship and couple functioning. Uncovering information relating specifically to this topic may be particularly relevant to try to understand the factors involved in the high divorce rates. Moreover, the patient’s condition and couple functioning may influence each other.

We therefore conducted a systematic literature search to evaluate the existing data on bipolar disorder and marital issues, with a special focus on the help and support that could be provided by mental health professionals in this regard. For the review, electronic searches of articles published between 1 January 1970 and 31 March 2021 were done using various search engines such as PubMed, Google Scholar, and Science direct. The search terms used in various permutations and combinations included bipolar disorder, marital relationship, couple relationship, couple functioning, partner, spouse, children and couple therapy. We excluded articles on caregivers’ issues, in which a proportion of caregivers were partners, but no separate analysis was done to compare the caregivers’ issues of partners and other caregivers. Articles on children of parents with bipolar disorder were considered only when they contained issues related to couple functioning. The literature search resulted in 27 articles included in the review. The results of the literature search were regrouped into six topics, which will be addressed in the following sections. In addition, we reported clinical material from three patients followed in our day hospital, to illustrate some of the issues addressed in this review.

## 2. The Negative Impact of Bipolar Disorder on Life Partners

The negative impact of bipolar disorder for partners includes self-sacrifice and caregiving burden, emotional impact, and health problems [12,13].

### 2.1. Self-Sacrifice and Caregiver Burden

Partners may give up their leisure time or work, have no time to think about themselves, with a life revolving around the patient. Caring the patient can become a full-time job. They frequently are the sole financial providers in the couple and have to take full responsibility for care of the house and children [12,13].

This may be illustrated by the following vignette:

Mrs. A., a 43-year-old married bipolar I woman, was referred for evaluation of protracted depression after she had stopped lithium treatment. She had a history of five previous hospitalizations, three for depression with suicide attempts and two for manic episodes. The first episode was a depressive episode with mixed features, which occurred in the post-partum period, after the birth of her daughter. During this episode she experienced her first panic attack and subsequently developed panic disorder with agoraphobia as well as social anxiety. She began to drink to cope with anticipatory anxiety and depressive symptoms. When she was put under lithium treatment, she gained weight and fought back against it by restricting feeding and vomiting. She experienced her second depressive episode while she was working as a bank employee, but afterwards decided to permanently stop working because she felt unable to deal with the pressure of high expectations. Her husband was a caring and hardworking salesman. After his wife’s first suicide attempt, he tried to limit business trips to avoid long absences from home. However, this became difficult when his wife stopped working because he wanted to maintain their standard of living. On Sundays, Mrs. A.’s husband particularly enjoyed driving his family to small country inns and having lunch with friends. This was no longer possible after Mrs. A. developed anxiety and eating disorders. Fearing that his wife could drive their daughter to school while being drunk, he decided to do it himself every day. He prepared dinners after work and ensured his wife took her medications. He also took medical appointments on her behalf.

### 2.2. Emotional Impact

The partners of bipolar patients may go through a wide range of emotions when facing the different aspects of the illness. At the outbreak of the illness, they are frequently perplexed and lost because they do not understand what is happening [12,13]. This was particularly the case for Mrs. A.’s husband when his wife became manic for the first time. He could not understand what was happening when she accused him to be the worst person in the world and to be responsible for everything wrong. He said he was totally unprepared for his wife’s considerable change. It was incomprehensible for him to see the mother of their child wearing sexually provocative outfits to go to work. During the course of the illness, there are a lot of reasons for partners to be frightened and experience a sense of anxiety. The risk of relapse, suicide, violence, and legal issues may induce a permanent threat for the partner [12,13]. After his wife’s first suicide attempt, Mr. A. said he was always on guard and that this watchful attitude was draining his strength. He also expressed doubts about his own capacity of discernment and handling of the situation.

The sum of burdens may lead to feelings of uncertainty, insecurity, powerlessness or loneliness, which can create anger and despair [13]. Another painful feeling concerns the experience of grief [13]. In the case of Mrs. A.’s husband, the grief was related to his daughter’s loss of security and stability as she was growing up beside her ill mother.

### 2.3. Health Problems

The burdens may also lead to a number of partners developing health problems of their own and increasing visits to their primary care physicians [13]. The most common problems may involve somatic symptom disorders with the occurrence of tension, muscular pain, tiredness or insomnia [13]. Mrs. A. herself recognized that her husband was sometimes close to burn-out and that his general health was getting poorer. When meeting him at medical appointments, he was frequently on the verge of tears. These episodes of burn-out were the only times in which he could begin to speak about divorce, because he still loved his wife and was against the principle of divorce. Sometimes this kind of state can lead to diagnosed depression [13].

## 3. The Negative Impact of Bipolar Disorder on Couple Relationship

The negative impact of bipolar disorder on couple relationship includes a weakening of the bond, stigmatization, dissatisfaction with sexual life, and lower rates of childbearing [2,12,13].

### 3.1. Weakening Relationship

The profound changes and unpredictability of the patient’s behavior often create volatility and insecurity in the relationship [12]. Living with a partner is based on a commitment that implies the presence of some kind of future representation of the relationship, that is, of some kinds of goals in terms of expectations, hopes, or future concerns [1].

Such a commitment may be compromised by the illness, affecting the partners’ ability to trust one another and bringing doubt about their ability to stay in the relationship over the long term [12,13]. Mrs. A.’s husband expressed this feeling when he said that the illness has closed the door to the future they had together envisioned.

### 3.2. Stigmatization

Changes induced by the illness may also lead to stigmatization and loss of a social life. The couple can be the subject of gossip among friends and neighbors [12,13]. Most of the time, friends stop contacting them. Partners may also decide to cut off relationships with former friends, in order to conceal their problems. In the case of Mrs. A., this happened after she had been sexually provocative towards a good friend’s husband during a dinner organized for the birthday of their child. The loss of social interactions induces a limitation in communication and socialization with others, leading to a feeling of loneliness and of being abandoned by them. This also contributes to undermining the identity of the couple [12,13]. Sometimes stigmatization may come from the partner himself. Another of our patients, Mr. B., was a 36-year-old bipolar II engineer, married to a lawyer who had a histrionic personality disorder. The couple had no children. When he was overloaded with work, he occasionally took illicit substances to have more energy to cope with stress. In the course of an argument they had during a ride, his wife told him she was afraid to be driven by a ‘junkie’. When he sometimes raised his voice during their arguments, she could threaten him to put him into custody, even though he was euthymic at that time.

### 3.3. Dissatisfaction with Sexual Life

Couples where one partner has a bipolar disorder have been shown to be less satisfied sexually than couples without psychiatric disorder, with satisfaction diverging between patients and their partners. Both hypersexuality of manic episodes and hyposexuality of depressive episodes seem to be disruptive to the partner’s sexual satisfaction, with this effect often persisting into inter-episodes periods [14]. This often leads to decreased frequency of sexual intercourses [15]. In the case of Mrs. A. and her husband, this decrease occurred after the third episode to amount to once monthly. In less severe forms of the illness, the lower frequency could be explained by differences in chronotypes among partners. Mr. B., whose chronotype was characterized by marked eveningness, stated a preference for having sex in the evening, which was not the case for his wife who showed a morning chronotype. The lack of physical intimacy may also contribute to weaken the bond [12]. Regarding side effects of psychotropic treatments, erectile dysfunction was the most common effect reported with antipsychotics, and was more severe with typicals than with atypicals [2]. For lithium, the most common effect was decreased sexual desire, though a few patients experienced increased desire [2]. With regards to lamotrigine, some patients experienced sexual dysfunction, while other patients reported improved sexual functioning [2]. Mr. B. was complaining of impotence when treated with lamotrigine, but impotence improved when sildenafil was added to his regimen. Altered menstrual cycles were also reported for antipsychotics and valproate [2].

### 3.4. Lower Rates of Childbearing

Bipolar patients have been reported to have lower fertility rates [2]. However, findings may be controversial, due to methodological flaws in the studies [2]. A large study from Sardinia evaluated the reproductive outcome of patients with bipolar disorder (*n* = 523) and compared the same with major depressive disorder (*n* = 1351). When the number of children of patients with affective disorder was compared with the general population data of Sardinia, patients had 17% fewer children. Overall fertility rates in patients with bipolar I disorder were lower than those with major depressive disorder [16]. Several factors, including the role of psychotropic drugs, have been incriminated to explain these lower rates [2]. As far as couple relationship is concerned, decreased frequency of sex and renunciation to have children because of the disorder may be the main reasons. In the case of Mrs. A., the husband was aware of the genetic risk and knew that the patient’s sister was treated for bipolar disorder. This was the same for Mr. B.’s wife who read a lot of papers on this topic and frequently asked us about the risk of giving birth to a child more likely to developing bipolar disorder. These worries impacted both the frequency and quality of intercourses. Interestingly in this regard, higher levels of education in the partner have been associated with an increased perception of mental illness stigma [4]. Educated partners were deemed to be more sensitive to rejection or status loss and may therefore fear that their children experience the same kind of stigma [4].

## 4. The Negative Impact of Parental Bipolar Disorder on Children 

Some studies have found a relationship between poor marital adjustment in the parents and disorders in the children of parents with an affective disorder [17,18,19,20,21,22]. Other studies indicate that marital conflicts, rather than marital adjustment, may be strongly associated with child maladjustment, especially when one of the parents suffers from a mental disorder [23,24].

Marital conflicts may prove to be even more detrimental for children of bipolar parents, since these children were found to show more distress than other children when observing a conflict between adults and seem to take longer to overcome their distress [25]. It has been suggested that quality of parenting could be the factor that makes the difference between a positive or a negative outcome in children of parents with bipolar disorder [26,27]. The presence of depressive symptoms in parents could lead to low-quality interactions with their children and even result in increased levels of emotional neglect [17]. A recent study [28], which explored the experiences of young children who have parents with bipolar disorder, found that children were able to experience negative emotions affiliated to their parents’ behaviors when unwell. These studies suggest that, in addition to genetic factors, nongenetic factors may contribute to the development of psychiatric illnesses in children of bipolar parents, in keeping with vulnerability-stress models of mental disorders [17]. The 11-year-old daughter of Mrs. A. was resentful against her mother when she was seeing her vomiting or driving while being drunk. This feeling increased when she realized that such behaviors were a matter of conflict between her parents.

## 5. The Negative Feedback on the Patients

The negative impacts of the illness on the partners, couple functioning, and children may turn out to all have a negative feedback on the patients themselves. These impacts create an additional source of stress for them. It is well known that high levels of expressed emotion may trigger relapses in bipolar patients [29,30]. Strong emotional changes experienced within the family are likely to fuel such expressions. Arguments for everyday life matters are more frequent and may be reinforced by a lack of dialogue and cooperation. Cases of bipolar patients have been reported whose mood was frequently cycling under the stress of a continually troubled marriage, but which normalized as long as they could live a relatively placid life [30]. When euthymic, patients may express self-blame over their past behaviors towards their partners or children, as well as a feeling of shame for their lack of closeness and intimacy. In addition, they often feel guilty not to be able to maintain a job position, to be a role model for their children, or because they are aware that they are a burden to their family [12]. Mrs. A. said that it could sometimes seem as if mother and daughter had swapped roles within the family.

## 6. Are There Positive Impacts of Illness for the Couple?

Positive impacts of the illness were reported for the couple [12,13]. These impacts can be regrouped into three categories: personal evolution, strengthening relationship, and new goals and ideals for living together.

### 6.1. Personal Evolution

This may involve an increased empathy and compassion toward others, a sense of resilience in dealing with life’s hardships, and acquiring skills for illness management [12,13]. Mrs. A.’s husband said that he had learnt to adapt his communication style. When his wife was in a crisis he realized it was better not to contradict her and to wait until she was calm. He chose his words carefully and attempted to be more subtle about how he made suggestions. He had also learnt to sort out their friends and distinguish between those they could rely upon and those who were not safe to get in touch with. According to Mrs. A., her illness had taught her how to fight for her sanity and try to stay healthy.

### 6.2. Strengthening Relationship

Standing side by side through hard times may sometimes lead to a growing commitment to one another, a deepening of the bond and an increasing trust [12,13]. This is particularly the case when partners are successful in managing the difficulties they are confronted with. For Mrs. A.’ husband, trust was related to the belief that his wife could remain stable and comply with the treatment. For Mrs. A., trust had to do with the fair appreciation for care, affection, and support she received during difficult moments. She also highly appreciated that her husband was continuing to display his love to her despite the illness and their difficulties.

### 6.3. New Goals and Ideals for Living Together

Owing to the knowledge and insight acquired through their experiences, partners may gradually accept to live with the illness and its consequences. This acceptance process can bring them peace of mind and be liable to initiate a reconciliation process, whereby partners are able to reconcile with life as it is [13]. This reconciliation opens the path to new goals and ideals for living together, involving awareness of what is important in life, appreciation of life here and now, and planning only for the immediate future [13]. Mrs. A.’s husband had progressively accepted the idea that his wife could do well sometimes only for short periods of time, but was still happy to be able to do enjoyable things together with their daughter during such moments. Through positive realism, partners may find a new foundation for courage and joy in the existing possibilities in their shared life [13].

## 7. Improving Couple Functioning When One of the Partners has Bipolar Disorder

To achieve this goal, one has to take into account two further sets of data:

(1) care experiences of partners of bipolar persons and (2) findings from couple therapy. Comprehensive approaches are also worthy of consideration.

### 7.1. Care Experiences of Partners of Bipolar Persons

Experiences that may be of particular concern here are those dealing with the relationship with health personnel and those about couple relationship during crisis management. Partners have a variety of complaints about the way they are handled by mental health professionals. They frequently complain of being overlooked or turned away by health personnel [13]. They feel they lacked care and information when the patient was hospitalized. After discharge, they often complain of no follow up from the health service, lack of advice, and not having been trained about how to manage difficult situations. Mrs. A.’s husband stated that, during the previous hospitalizations of his wife, professionals did not encourage his involvement and dismissed his concern. There had been a good deal of learning by doing in this field. Only when he realized that it was not his wife who was responsible for what could happen but the illness itself, he was able to try more. This shows that when the disturbance is clearly recognized by the partner as an illness, then that partner is usually more tolerant of further episodes. In case of a crisis, many partners have experienced what has been called the ‘dilemma of help’ [31], the dilemma of whether to risk the situation escalating, or act without the patient’s knowledge, which could damage the couple’s relationship. Another among our patients, Mr. D., was a 41-year-old university surgeon who was suffering from a bipolar I disorder associated with cocaine use disorder. He was married to a dermatologist who had an alcohol use disorder. Both were working in the same hospital. At the outset of Mr. D.’s second manic episode, his wife told him he was overly excited and needed psychiatric care. Mr. D. answered that she was probably drunk and that, if he had been mad, he would have strapped her on the kitchen table and cut her into pieces. As she was terrified hearing this, she called the police and a psychiatrist who was a friend of theirs. Mr. D. was brought into the emergency department of his hospital and committed to the psychiatry service. After the episode, he decided to divorce and to sue the psychiatrist.

In order to prevent such situations, many partners, patients, and healthcare professionals have expressed the desire for better communication between them, and a shared crisis plan. Shared agreement may reduce anxiety and prevent crises from escalating [32].

Attitudes to adopt towards bipolar persons is a matter of great concern among partners [32]. Patients may experience efforts of their partners to prevent relapse as too intrusive, or respond angrily to suggestions about illness management. Not all partners may find, on the sole basis of their own experience, the best way to communicate with the patient. It was found that a certain level of emotional disclosure could be useful and help motivate behavior change [32]. Mrs. A. reported that having had a frank discussion with her daughter about her vomiting behavior made their relationship more intimate, which positively impacted the bond with her husband.

To summarize, experiences of partners reveal a certain number of unmet needs concerning communication with professionals, information given to them, and specific education especially on how to adapt their relational style to increase patient receptivity to their input [13,32].

### 7.2. Findings from Couple Therapy

Literature on the effectiveness of marital therapy on couple functioning and illness outcome in bipolar disorder is scarce. A first study [33] compared patients receiving medication management only following their index hospitalization, patients who were referred back to their home community for care following hospital discharge, and patients receiving both medication management and weekly couples’ group psychotherapy as post hospital treatment. All these bipolar patients were also treated with lithium. The drug-plus-psychotherapy group was found to have fewer major life disruptions and better social functioning compared to the two other groups. In a second study [34], the partners of bipolar patients attending psycho-education sessions were compared with controls. After the sessions and 6-month later they showed more knowledge of the disease, medications, and social strategies. The partners highlighted the fact that they had shared their problems with others or had gained a better understanding of the illness. However, patient compliance did not change over the next years. In a third study [35], patients were randomly assigned to receive either medication management or medication management plus a marital intervention with their spouses. Significant effects favoring the combined treatments were observed for overall patient functioning but not for symptoms levels. In this study, the marital intervention was associated with improved medication adherence. Unfortunately, these studies may be skewed by the presence of methodological flaws such as the small number of patients included, the comparability of the different groups or the age of population studied, and the lack of specifically-designed and standardized programs for marital interventions.

### 7.3. Comprehensive Approaches

According to these approaches, security and stability in a shared life can be perceived as the presence of equilibrium in subjective and relational shared life experiences, as oneself, the partner, and the shared life relationship were a complete gestalt, aka meaningful whole [13]. A complete gestalt is seen as a holistic experience, made possible by the fact that the person has insight into, and finds meaning in the individual elements that together make up the whole [13]. Complete gestalts contribute to the equilibrium of the human organism; when only parts of this whole are experienced, an incomplete gestalt is formed in the person’s lived experience. This counteracts the equilibrium and creates imbalance in the person. Furthermore, the degree of insight and meaning partners derive from an experience affects whether this experience may be perceived as a complete or incomplete gestalt [13]. With the outbreak of bipolar disorder, a series of unexpected experiences emerge from which people are unprepared to derive meaning, affecting their ability to cope with them [13]. The aim of couple therapy may therefore be viewed as empowering partners to derive insight, meaning, and a holistic understanding of their experiences with bipolar illness. It was suggested that this goal could be achieved with interventions mixing psychoeducation on bipolar illness, care, health-promoting education, and guidance dialogues through which partners could work through their thought processes and emotional reactions [13].

## 8. Conclusions and Future Directions

This review shows that having a marital relationship with a bipolar person has an impact on the partners’ life, their state of health, as well as the relationship itself. The illness also has consequences on the sexual life of the couple, their children, and their fertility rates. Impacts are mainly negative, but sometimes can be positive. All this may, in turn, have consequences on the illness itself.

The findings of the current study may be relevant in at least three domains: care, psychiatric research, and sociology.

With regards to care, health professionals have to take seriously into account the complaints and demands of the partners, adopt empathetic attitudes, provide them with education and support, empower them as an effective therapeutic influence, as well as help them in bringing insight and meaning to their experiences. This may contribute to restoring a relation of trust between patient and partner, which can be a useful tool in the management of the illness. Taken as a whole, interventions should be aimed at minimizing the negative impacts of the illness while favoring the positive ones. According to the clinical experience of some authors [36], it should also be wise, in order to help prevent divorce and first of all emotional divorce, to involve the partner as early as possible, before anger or bitterness at all the disruptions become intolerable. With respect to research, there is a need for well-designed, large sample size, randomized controlled studies on the effectiveness of couple therapy in the context of bipolar disorder. These studies should also take into account the impact of comorbid conditions and bipolar subtypes, as suggested by previous research [11] and our clinical cases. Among the different domains and variables of marital life impacted by the illness, research should sort out those which might be preferentially targeted for therapeutic interventions to improve the illness course. Interestingly, it was found in a recent study [37] that problem areas in marital life were differing between male and female partners of bipolar patients. Standardized instruments designed to assess the subjective burden of partners as caregivers [38] or the quality of marital relationship [39] could be useful for such research. In addition, the effect of marital interventions on children’s quality of life, psychopathology, and long-term outcome should be worthy of investigation. The issue as to whether, in the context of bipolar disorder, couples who have children are less likely to divorce than those who have not, has not been addressed so far and may deserve further studies.

More systematic studies on partner’s psychopathology are also necessary. In the case of Mr. B., the psychopathology of his wife preceded the outbreak of his own illness, whereas the reverse order was true for Mr. D. The order of onset and the interplay of partners’ disorders might be worthy of investigation when one of these partners suffers from bipolar disorder, as suggested by previous research as well [40,41].

Finally, it was shown that divorce impacts more than the individuals who choose to end their marriage, which takes a toll on society [42]. Divorce has a far-reaching impact on families’ financial livelihoods, children, and the workplace. The societal impact of divorce in the case of bipolar disorder may also deserve, in this regard, scientific studies.

## Data Availability

Due to data protection laws, we are not able to provide the clinical data of the patients.
